# Use of Radical Oxygen Species Scavenger Nitrones to Treat Oxidative Stress-Mediated Hearing Loss: State of the Art and Challenges

**DOI:** 10.3389/fncel.2021.711269

**Published:** 2021-09-01

**Authors:** Isabel Varela-Nieto, Silvia Murillo-Cuesta, Lourdes Rodríguez-de la Rosa, María Jesús Oset-Gasque, José Marco-Contelles

**Affiliations:** ^1^Institute for Biomedical Research “Alberto Sols,” Spanish National Research Council (CSIC)-Autonomous University of Madrid, Madrid, Spain; ^2^Biomedical Research Networking Center on Rare Diseases (CIBERER), Institute of Health Carlos III, Madrid, Spain; ^3^Hospital La Paz Institute for Health Research, Madrid, Spain; ^4^Department of Biochemistry and Molecular Biology, School of Pharmacy, Complutense University of Madrid, Madrid, Spain; ^5^Institute of Neurochemistry Research, Complutense University of Madrid, Madrid, Spain; ^6^Laboratory of Medicinal Chemistry, Institute of General Organic Chemistry, CSIC, Madrid, Spain

**Keywords:** antioxidants, free radicals, sensorineural hearing loss, *N*-acetyl-L-cysteine, NXY-059, 4-OHPBN, PBN

## Abstract

Nitrones are potent antioxidant molecules able to reduce oxidative stress by trapping reactive oxygen and nitrogen species. The antioxidant potential of nitrones has been extensively tested in multiple models of human diseases. Sensorineural hearing loss has a heterogeneous etiology, genetic alterations, aging, toxins or exposure to noise can cause damage to hair cells at the organ of Corti, the hearing receptor. Noxious stimuli share a battery of common mechanisms by which they cause hair cell injury, including oxidative stress, the generation of free radicals and redox imbalance. Therefore, targeting oxidative stress-mediated hearing loss has been the subject of much attention. Here we review the chemistry of nitrones, the existing literature on their use as antioxidants and the general state of the art of antioxidant treatments for hearing loss.

## Introduction

**FIGURE 1:**
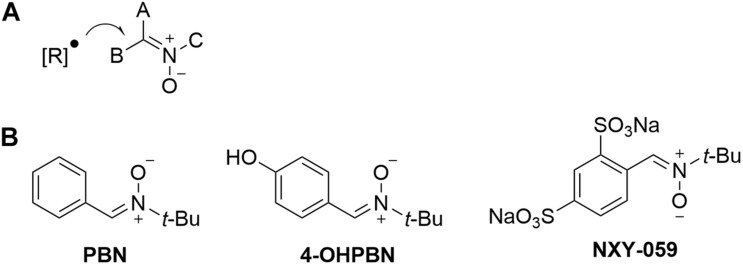
**(A)** Reaction of a free radical [R]^∙^ with a nitrone. **(B)** Structures of nitrones PBN, 4-OHPBN and NXY-059.

Nitrones are organic molecules able to trap reactive oxygen and nitrogen species (ROS, RNS) (Rosselin et al., 2017). Thus, nitrones constitute potent antioxidant molecules able to reduce oxidative stress (Firuzi et al., 2011). Nitrones power to scavenge free radicals derives from its activated carbon nitrogen double bond (Figure 1A) that prompts easy free radical attack leading to less reactive and harmful nitroxide species for biomolecules (Oliveira et al., 2018). However, the fact that the doses used for spin trapping experiments are 1,000-fold higher than those usually applied in the *in vitro* neuroprotection assays (10–50 μM), and that the amounts of nitrones used *in vivo* are currently under 50 μM, clearly insufficient to trap ROS/RNS, suggest that other mechanisms are responsible for nitrones scavenging capacity (Rosselin et al., 2017). Nitrones also suppress signal transduction processes with significant anti-inflammatory, anti-apoptotic (Floyd et al., 2008) and NO-releasing properties (Croitoru et al., 2011). These actions together with ROS and RNS scavenging may account for the antioxidant/neuroprotective profile exhibited by nitrones (Villamena et al., 2012).

Nitrone derivatives have been extensively used in both preclinical models and clinical assays as therapeutics to reduce oxidative stress in several pathologies, mainly stroke (Marco-Contelles, 2020), neurodegenerative disorders (Cancela et al., 2020) and cancer (Thomas et al., 2020). Oxidative stress is also considered a major pathological mechanism in several auditory pathologies, including ototoxicity, noise-induced hearing loss (NIHL) and presbycusis (Bermúdez-Muñoz et al., 2020, 2021; Varela-Nieto et al., 2020).

Thus, the purpose of this minireview is to discuss how the antioxidant properties of selected nitrones, such as alpha-phenyl-tert-butylnitrone (PBN), 4-hydroxy PBN (4-OHPBN), disufenton sodium (NXY-059, also referred to as HPN-07) (Figure 1B), have been explored as potential therapeutic small molecules for the treatment of hearing loss of different etiologies and to identify the current challenges to translate these results into the clinical practice.

## Nitrones as Therapeutic Agents for Sensorineural Hearing Loss

Sensorineural hearing loss (SNHL) is the most common sensory deficit in adults, and is associated with normal aging. SNHL is intensified due to the exposure to pollutants such as carbon monoxide (CO) (Marcano Acuña et al., 2019), hydrogen cyanide (Fechter et al., 2002), trimethyltin (Yu et al., 2016) and acrylonitrile, as well as by the combination of high levels of noise and these toxins (Fechter et al., 2004; Pouyatos et al., 2009). SNHL is also caused by drugs such as aminoglycosides and cisplatin (Kros and Steyger, 2019; Murillo-Cuesta et al., 2021). Although the underlying mechanisms of age- or environmental toxins- mediated SNHL are still unknown, there is large consensus in that free radical processes are effectively involved (Fechter et al., 2004; Wang et al., 2007; Fetoni et al., 2019; Varela-Nieto et al., 2020).

Oxidative stress plays a key role in the induction of cochlear injury after noise exposure (Wang et al., 2007; Varela-Nieto et al., 2020). Very interestingly, one of the consequences of the oxidative stress induced by excessive noise is the onset of apoptosis linked to mitochondrial release of cytochrome C, activation of caspases and the N-terminal-c-JUN kinase pathway (Wang et al., 2007).

The key role of oxidative stress in producing cochlear injury is supported by the positive therapeutic effect shown by antioxidants in preclinical studies carried out in animal models. Furthermore, deletion of antioxidant enzymes like *Gpx1* (glutathione-peroxidase1) results in NIHL and loss of hair cells in mice (Ohlemiller et al., 2000). Accordingly, the organoselenium compound Ebselen, which mimic GPX1 activity, prevents outer hair cells loss in rats after oral treatment, before and immediately after noise exposure (Kil et al., 2007). Ebselen has also shown a moderate effect in clinical trials for NIHL (Kil et al., 2017). Also, adenoviral-mediated antioxidant gene therapy to overexpress human catalase and superoxide dismutases (SOD1 and SOD2) has been used to prevent aminoglycoside ototoxic trauma in the guinea pig cochlea (Kawamoto et al., 2004). Supplementary Table 1A summarizes main published data studying antioxidants, such as acetyl-*L*-carnitine (ALCAR), *N*-acetyl-L-cysteine (NAC) or Ebselen, among others. NAC and ALCAR are known to efficiently reduce NIHL (Choi, 2011; Varela-Nieto et al., 2020) and aminoglycoside ototoxicity (Somdas et al., 2015; García-Alcántara et al., 2018). Furthermore, NAC also reduces oxidative stress, inflammation and protects hearing (Bermúdez-Muñoz et al., 2021) in a genetic model of early onset age-related hearing loss (Celaya et al., 2019). More recently, researchers have tested HK-2, a multifunctional antioxidant, which has been shown to protect against NIHL and hair cell loss administered orally in rats before and after noise (Chen et al., 2020).

Despite their potent antioxidant power, nitrones have been poorly studied as a therapy for SNHL. Alone or in combination with other well-known antioxidant agents, such as NAC (Kopke et al., 2007, 2015), nitrones have been reported to be effective in the prevention and treatment of NIHL (Kopke et al., 2005; Ewert et al., 2017) or CO ototoxicity (Fechter et al., 1997).

Here we will discuss in detail the reported actions of free radical scavenger nitrones PBN, 4-OHPBN and NXY-059 (Figure 1B) in SNHL protection.

### PBN

Phenyl-tert-butylnitrone (Figure 1B) has been shown to be effective in mitigating the SNHL that occurs in Long Evans hooded rats exposed to CO (Rao and Fechter, 2000) or to acrylonitrile (Fechter et al., 2004), plus exposure to high level noise. Acrylonitrile when associated with acoustic overexposure increased hearing loss, implying that the mechanism of the potentiation of NIHL by these compounds involved enhancing cochlear oxidative stress (Pouyatos et al., 2005). Rao and Fechter observed that PBN given before and after high-level steady-state noise decreased CO-mediated potentiation of the noise-induced threshold shifts (Rao and Fechter, 2000). However, no statistically significant differences were found between animals only exposed to noise and untreated or treated with PBN (Rao and Fechter, 2000). The underlying mechanisms of PBN have not been studied in detail, but may involve the reduction of oxidative stress (Floyd, 1997). Indeed, PBN protects cochlear function from combined exposure to noise and CO by reducing the formation of ROS/RNS in the cochlea (Fechter et al., 2000). The otoprotection shown by PBN on acrylonitrile-induced damage seems due to oxidative stress reduction by preventing the depletion of glutathione caused by combined noise and acrylonitrile exposure and also reducing reactive epoxide binding to cytochrome C oxidase (Pouyatos et al., 2005). PBN was reported to have a potential key role in increasing the Cu/Zn form of superoxide dismutase in the cochlea (Pierson and Gray, 1982), which has been shown in other organs to restore the same type of antioxidant defense mechanisms associated with cochlear protection (Weisiger and Fridovich, 1973).

### 4-OHPBN

Nitrone 4-OHPBN (Figure 1B), a PBN derivative, and its major metabolite, has been reported to decrease permanent NIHL in chinchilla (Choi et al., 2008). This was very interesting because a previous report claimed that PBN was ineffective in reducing noise-induced auditory threshold shifts (Rao and Fechter, 2000). In fact, 4-OHPBN, administrated after 4 h of noise exposure, proved effective in the treatment of NIHL, and when administrated in combination (4-OHPBN + NAC, and 4-OHPBN + NAC + ALCAR) with other antioxidant drugs such as NAC or ALCAR, showed increased efficacy, since each antioxidant targeted different injury mechanisms (Choi et al., 2008). Choi et al. (2008) reported that animals exposed to a 105 dB octave-band noise for 6 h and then treated with 4-OHPBN drug combinations for 4 h increased the efficiency of the treatment, reducing the individual drug dose. The precise mechanisms by which 4-OHPBN reduces cochlear injury are still largely unknown; among the targets proposed by different authors we found free radical scavenging, inhibition of iNOS activation, suppression of ROS/RNS formation, decreased mitochondrial ROS production, reduced neuroinflammation and activation of MAP kinase cascades (Floyd, 1999; Du et al., 2011).

### NXY-059

The potential therapeutic effect of nitrone NXY-059 (Figure 1B) and of its combination with NAC to treat permanent NIHL was explored in female chinchillas exposed to a 105 dB octave-band noise centered at 4 kHz, for 6 h, by starting treatment 4 h after noise exposure and continually injecting twice daily for the next 2 days (Choi, 2011). Results showed that the mean permanent hearing threshold of NXY-059 and NXY-059 + NAC treated groups was decreased compared to the noise exposed group. Furthermore, NXY-059 + NAC showed greater effects, demonstrating that this drug combination enhances the therapeutic effect (Choi, 2011). Indeed, the combination of antioxidants with different and complementary mechanisms of action has proven to be more effective than using just one drug (García-Alcántara et al., 2018). NXY-059 + NAC has been further evaluated as a therapeutic approach for NIHL in rats exposed to 115 dB octave-band noise (10–20 kHz) treated 1 h before and 1 h after noise exposure, and then for two consecutive days (Lu et al., 2014). Auditory brainstem response showed that this treatment significantly reduced the threshold shift across all tested frequencies along the 21 days studied. Reduced distortion product otoacoustic emission level shifts were also detected at 7 and 21 days following noise exposure of treated animals. Protection was associated to increased conservation of outer and inner hair cells in the organ of Corti. Treatment also significantly reduced the noise-induced expression of c-FOS in the cochlear nucleus neurons. These results indicated that NXY-059 + NAC is a promising pharmacological combination for NIHL therapy, as it decreases both temporary and permanent threshold shifts after intense noise exposure, protects cochlear sensory cells, and potentially afferent neurites, from the damaging effects of noise-induced oxidative stress. In addition, the drugs reduced aberrant activation of neurons in the central auditory regions of the brain following noise exposure (Lu et al., 2014). Finally, NXY-059 free radical spin trapping activity has been reported to protect the cochlea exposed to acute acoustic trauma (Ewert et al., 2017).

## Conclusion and Future Perspectives

The pathophysiological response to oxidative stress has been a widely studied and is a well-established cochlear mechanism of injury leading to hearing loss (Choi and Choi, 2015; Wang and Puel, 2018; Bermúdez-Muñoz et al., 2020, 2021; Varela-Nieto et al., 2020). Oxidative stress occurs when cochlear cells show excessive amounts of oxidants or decreased levels of antioxidants and causes formation of free radicals. These free radicals can damage cellular DNA, proteins, lipids, and unregulated apoptotic pathways, causing cell death and irreversible damage to the cochlea (Kurabi et al., 2017). Secondary to oxidative stress, inflammation occurs and further expands cochlear injury leading to apoptotic cell death of the irreplaceable sensory hair cells and neurones (Perin et al., 2021). In this context, nitrones have been explored to prevent hearing loss rendering modest positive results, generally combined with NAC. The enormous potential of these molecules invites to study in depth their largely unknown biodistribution and pharmacokinetics when administered by different routes to the inner ear. Improving their solubility in biological membranes by means of medical chemistry strategies, the modification of their structure or their combination with biocompatible vehicles, could allow their local administration, which could eventually improve their preclinical results.

## Author Contributions

IV-N, SM-C, and JM-C wrote the manuscript. LR and MO-G revised the manuscript. All authors revised and approved the manuscript.

## Conflict of Interest

The authors declare that the research was conducted in the absence of any commercial or financial relationships that could be construed as a potential conflict of interest.

## Publisher’s Note

All claims expressed in this article are solely those of the authors and do not necessarily represent those of their affiliated organizations, or those of the publisher, the editors and the reviewers. Any product that may be evaluated in this article, or claim that may be made by its manufacturer, is not guaranteed or endorsed by the publisher.
