# Supplementary material for: Use of Radical Oxygen Species Scavenger Nitrones to Treat Oxidative Stress-Mediated Hearing Loss: State of the Art and Challenges
Source: Front Cell Neurosci. 2021 Sep 1;15:711269. doi: 10.3389/fncel.2021.711269 (PMC8440819; doi:10.3389/fncel.2021.711269)
Supplement: Supplementary file 1 [file Table_1.docx]

**Supplementary Table 1**

**A**

| **Drug** | **HL** | **Spp** | **Posology** | **Main outcome** | **Reference** |
| --- | --- | --- | --- | --- | --- |
| ALCAR | ARHL | R | 50 mg/kg/d/30 day/PO | Significant ↓ of ABR latencies | Derin et al., Clin Otolaryngol Allied Sci. 2004 Jun;29(3):238. |
|  | NIHL | CH | 100 mg/kg/IP (BID 2 days before + 1 h prior to + 1 h after + BID 2 day after noise [155 dB SPL, 150 repetitions]). | Significant ↓ of permanent TS and HC loss | Coleman et al., Hear Res. 2007 Apr;226(1-2):104. |
|  | AG | GP | 100 mg/kg/day in water, starting 2 weeks before or simultaneously with gentamicin (100 mg/kg/day/7 days) | ↓ Hearing threshold and cochlear damage in new-born GPs exposed to gentamicin in utero | Kalinec et al., Proc Natl Acad Sci U S A. 2005 Nov 1;102(44):16019. |
|  | CPT | R | 200 mg/kg/SC 30 min before CPT (16 mg/kg) IP infusion | ↓ Auditory impairment, ↑ antioxidative and antiapoptotic parameters. | Gunes et al., Chemotherapy. 2011;57(3):186. |
| MET | NIHL | CH | 200 mg/kg/IP/ (BID 2 days before + 1 h prior to + 1 h after + BID 2 days after 6 h 105 dB SPL 4 kHz octave band noise | Significantly ↓ noise-induced TS and mean HC loss | Kopke et al., Laryngoscope. 2002 Sep;112(9):1515. |
|  | CPT | R | D-methionine (300, 350 and 400 mg/kg/IP) + CPT (14 mg/kg) | Complete auditory preservation with 350 mg/kg MET | Lorito et al., Med Sci Monit. 2011 Aug;17(8):BR179. |
| NAC | ARHL | M | 10 g/l in drinking water from weaning until 6 months of age | ↓ Hearing deficits and IHC loss | Ding et al., Aging (Albany NY). 2016 Apr;8(4):730. |
|  | NIHL | CH | 325 mg/kg (BID 2 days before + 1 h prior to + 1 h after + BID 2 day after noise [155 dB SPL, 150 repetitions]) | ↓ PTS and HC loss compared to saline-treated animals | Ding et al., Aging (Albany NY). 2016 Apr;8(4):730. |
|  | AG | ZE | 25–200 μg/ml; 0.15–1.23 mM) for 30 min + 20 μM Neomycin | ↓ ROS production in neuromasts exposed to neomycin | Wu et al., J Appl Toxicol. 2015 Mar;35(3):273. |
|  | CPT | GP | 250 mg/kg/IP or trans-tympanic (2%) before by CPT (5.5 mg/kg/day/3 days/SC) | ↑ Otoprotection | Mohan et al., Otolaryngol Head Neck Surg. 2014 Nov;151(5):836. |
| GSH | NIHL | CH | 50, 100 or 150 mM IT 40 min before noise (single 145 dB SPL impulse or continuous 105 dB SPL, 4 kHz octave band noise) | Significant protection from impulse, but not from continuous noise | Hight et al., Hear Res. 2003 May;179(1-2):21 |
|  | AG | GP | PO immediately prior to gentamicin injection (100 mg/kg/day/2 weeks) | Significantly ↓ the TS by 20-40 dB and ↓ HC loss | Garetz et al., Hear Res. 1994 Jun 15;77(1-2):81. |
|  | CPT | R | GSH or GSH ester (0.5, 1, 1.5 g/kg)/IP 30 min prior CPT 16 mg/kg/IP | 500 mg/kg GSH ester ↓ HL and outer HC loss. Protection decreased as dosage increased | Haim et al., Comp Biochem Physiol A Comp Physiol. 1985;80(4):50. |
| EBS | NIHL | R | 4 mg/kg/PO/12h for 3-14 day before and after noise | Significantly ↓ TS at all frequencies, outer HC loss and strial swelling | Kil et al., Hear Res. 2007 Apr;226(1-2):44. |
|  | CPT | R | Alone or combined with allopurinol + CPT (16 mg/kg/IP) | Significant protection to the cochlea and kidney from CPT toxicity. | Lynch et al., Hear Res. 2005 Mar; 201(1-2):81. |
| FA | AG | M | 50 mg/kg gentamicin + 100 mg/kg FA in 0.5 mL normal saline. | Restored ABR TS and DPOAE in gentamicin-induced HL. | Gu et al., Exp Cell Res. 2017 Nov 15;360(2):257. |
|  | CPT | R | 75, 150, 300 and 600 mg/kg 1 h before CPT (16 mg/kg/IP infusion) and SID for the following 3 d. | Up-regulation of Nrf-2/HO-1 and down-regulation of p53 phosphorylation pathways | Paciello et al., Sci Rep. 2020 Jan 23;10(1):1063. |
| RA | NIHL | R | IT (20 μl) or systemic (10 mg/kg, 1 h before noise exposure and SID for the following 3 days) | Significantly ↓ NIHL and cochlear oxidative stress | Fetoni et al., Hearing Loss. Otol Neurotol. 2018 Mar;39(3):378. |
|  | CPT | M | 4 mg/kg/IP, 4 consecutive days before CPT (2 mg/kg/IP for 8 days) | ↓ ABR thresholds | Jeong et al., PLoS One. 2011 Apr 15;6(4):e18815. |
|  | CPT | M | 100 mg/kg/IP/2 days before or after CPT 20 mg/kg | ↓ ROS accumulation and apoptotic cell death | Kim et al., Cell Death Dis. 2018 Aug 1;9(8):827. |
|  | AG | M | 75 mg/kg/12h/14 days/SC + kanamycin (750 mg/kg/SC) | ↓ ABR TS, decreased p38 MAPK and JNK | Wang et al., Neural Regen Res. 2012 Dec 15;7(35):2793. |
|  | NIHL | GP | 200 mg/kg/PO 24 h before and 72 h after noise (176 dB SPL, 1.05–20.3 kHz). | Significantly ↓ ABR deficits, HC damage and malondialdehyde concentration | Xiong et al., Clin Med Insights Ear Nose Throat. 2012 Nov 29;5:25. |
| NIT | NIHL | CH | NXY-059 four h after acoustic trauma (4 kHz octave band noise, 105 dB SPL 6h) | Enhanced auditory functional recovery, ↓ of HC, afferent nerve fibers and fibrocyte loss | Ewert et al., PLoS One. 2017 Aug 23;12(8):e0183089. |
| Vit | ARHL | M | Supplemented diet (L-cysteine-GSH mixed disulphide, ribose-cysteine, NW-nitro-L-arginine methyl ester, folate, Vit B12, C) | ↓ ABR TS. | Heman-Ackah et al., Otolaryngol Head Neck Surg. 2010 Sep;143(3):429. |
|  | NIHL | GP | 100 mg/kg/PO Vit E 24 h before and 72 h after exposure to impulse noise (176 dB SPL, 1.05–20.3 kHz) | Significantly ↓ ABR deficits, hair cell damage, and MDA concentrations. | Xiong et al., Ear Nose Throat. 2012 Nov 29;5:25. |
|  | AG | GP | Diet supplemented with β-carotene, vit C and E, and Mg 10 d + daily gentamicin injections for 16 d | Significant ↓ thresholds at frequencies ≤ 12 kHz. Robust protection of hair cell populations. | Le Prell et al., J Assoc Res Otolaryngol. 2014 Jun;15(3):375. |
|  | CPT | R | 100 mg/ml IT vitamin C 30 min before CPT 16 mg/kg/IP | Protective effect primarily at 2 kHz | Celebi et al., Eur Arch Otorhinolaryngol. 2013 Mar; 270(4):1293. |
|  | NIHL | R | Noise (10 kHz, 100 dB SPL 60 min during 10 d) + Q-Ter (100 mg/kg or 10 mg/kg CoQ10)/IP. | ↓ oxidative-induced cochlear damage, HL, and cortical dendritic injury. | Fetoni et al., J Neurosci. 2013 Feb 27;33(9):4011. |
| Q-Ter | AG | GP | Gentamicin (100 mg/kg/IV) + Q-Ter (100 mg/kg/IP) for 14 consecutive days. | ↓ the progression of HL, yielding a TS of 20 dB and the loss of outer HCs | Fetoni et al., Acta Otorhinolaryngol Ital. 2012 Apr;32(2):103. |
|  | CPT | R | CPT (14 mg/kg/IP or 4.6 mg/kg/3 days) + Q-Ter PO for 5 days. | Prevented and completely neutralized ototoxicity in rats treated with three daily CPT doses | Astolfi et al., PLoS One. 2016 Sep 15;11(9):e0162106. |
|  | ARHL | M | 4 g/kg/day dietary supplementation for 10 months | Significantly ↓ age-related auditory TS and HC loss | Pang et al., Neurobiol Aging. 2019 Aug;80:127. |
| RESV | NIHL | M | 4 g resveratrol/kg in chow for 2 months + noise (10-kHz, 120 dB SPL, 1 h) | ↑ cochlear SIRT1 activity, better recovery of hearing, ↓ HC loss and oxidative stress in the cochlea after noise | Xiong et al., Audiol Neurootol. 2017;22(4-5):303-310. |
|  | AG | R | Resveratrol (10 mg/kg) + NAC (400 mg/kg) for 5 days. On day 2, kanamycin + furosemide on the round window. | ↓ TS at high frequencies (≈10 dB), Protective effect fades after the cessation of the treatment | García-Alcántara et al., Hear Res. 2018 Feb;358:10. |
|  | CPT | R | 10, 1 or 0.1 mg/kg/day/PO for 10 d + CPT 16 mg/kg on day 11 | 10 and 1 mg/kg/d doses ↓ ototoxicity | Olgun et al., Laryngoscope. 2014 Mar;124(3):760. |
|  | ARHL | R | 100 mg/kg/d of EGb761 in water for 4 or 12 months | Significantly ↓ age-related caspase 3/7 induction along with significant improvements of ASSR TS. | Nevado et al., Acta Otolaryngol. 2010 Oct;130(10):1101. |
| GB | NIHL | R | 100 mg/kg/d/PO for 21 d. Exposure to white noise at 100 dB SPL for 8 h at the beginning of the study. | Significant ↓ in ABR thresholds 1 d after noise. ↓ IL-1 Beta, IL-6, TNF-alpha and COX-2 cochlear expression. | Dogan et al., Auris Nasus Larynx. 2018 Aug;45(4):680. |
|  | AG | GP | Feeding with 100 mg/kg/d for 2 days. On the third day, IT injection of gentamicin (50 μl of 40 mg/ml) | Significantly ↓ ROS/NO production and HC apoptosis. | Yang et al., J Nutr Biochem. 2011 Sep;22(9):886. |
|  | CPT | R | 100 mg/kg/d/PO for 10 d + CPT 12 mg/kg/IP | Significant ↑ in the DP-grams | Esen et al., J Int Adv Otol. 2018 Apr;14(1):22. |
|  | ARHL | M | ω3-supplemented diet, ad libitum for 8 month | Protection of cochlear metabolism and HL progression | Martínez-Vega et al., J Nutr Biochem. 2015 Dec;26(12):1424. |
| PUFAS |  |  |  |  |  |

**B**

| **Drug** | **HL** | **Code NCT** | **Title** | **Age** | **Gender** |
| --- | --- | --- | --- | --- | --- |
| MET | NIHL | 02903355 | Phase 3 Clinical Trial: D-methionine to Reduce Noise-Induced Hearing Loss | 21-45 | All |
| NAC | NIHL | 00552786 | Antioxidant Medication for Noise-induced Hearing Loss | 25-65 | Male |
| NAC | AG | 01271088 | Protective Effect of N-acetylcysteine Against From Ototoxicity | 18-65 | All |
| NAC | NIHL | 01727492 | Prevention of Noise-induced Damage by Use of Antioxidants | 18-25 | All |
| NAC | CPT | 02094625 | NAC to Prevent Cisplatin-induced Hearing Loss | 1-21 | All |
| NAC | CPT | 03400709 | Protective Role of N-acetylcysteine From Cisplatin-induced Ototoxicity in Patients With Head and Neck Cancer | >18 | All |
| NAC | CPT | 04226456 | Intratympanic Administration of N-acetylcysteine for Protection of Cisplatin-induced Ototoxicity | >18 | All |
| NAC | CPT | 04291209 | Intratympanic N-Acetylcysteine for Prevention of Cisplatin-induced Ototoxicity. | >18 | All |
| STS | CPT | 00652132 | A Multi-centre Open-label Randomised Phase III Trial of the Efficacy of Sodium Thiosulphate in Reducing Ototoxicity in Patients Receiving Cisplatin Chemotherapy for Standard Risk Hepatoblastoma | <18 | All |
| STS | CPT | 00716976 | Sodium Thiosulfate in Preventing Hearing Loss in Young Patients Receiving Cisplatin for Newly Diagnosed Germ Cell Tumor Hepatoblastoma, Medulloblastomas Neuroblastoma, Osteosarcoma or Other Malignancy | 1-18 | All |
| STS | CPT | 01369641 | The Effect of Sodium Thiosulfate Eardrops on Hearing Loss in Patients Who Receive Cisplatin Therapy | >18 | All |
| STS | CPT | 02281006 | Randomized Controlled Trial to Test the Efficacy of Trans-tympanic Injections of a Sodium Thiosulfate Gel to Prevent Cisplatin-induced Ototoxicity in Patients With Head and Neck Cancer | >18 | All |
| STS | CPT | 04262336 | Study to Evaluate Safety and Efficacy of DB-020 to Protect Hearing in Patients Receiving Cisplatin for Cancer Treatment | >18 | All |
| EBS | NIHL | 01444846 | Otoprotection With SPI-1005 for Prevention of Temporary Auditory Threshold Shift | 18-31 | All |
| EBS | CPT | 01451853 | SPI-1005 for Prevention and Treatment of Chemotherapy Induced Hearing Loss | 19-80 | All |
| EBS | CPT | 01452607 | Study to Evaluate the Safety and Pharmacokinetics of SPI-1005 | 19-50 | All |
| EBS | NIHL | 02779192 | A Phase 2b Study of SPI-1005 to Prevent Acute Noise Induced Hearing Loss | 18-50 | All |
| EBS | AG | 02819856 | SPI-1005 for Prevention and Treatment of Tobramycin Induced Ototoxicity | >18 | All |
| LA | CPT | 00477607 | Alpha-Lipoic Acid in Preventing Hearing Loss in Cancer Patients Undergoing Treatment With Cisplatin | >18 | All |
| NIT |  | 02259595 | Study to Determine the Safety, Tolerability, and Pharmacokinetic Profile of NXY-059 and NXY-059 + NAC | 18-55 | All |
| VA | NIHL | 02257983 | Protective Effects of EPI-743 on Noise-Induced Hearing Loss | 18-30 | All |
| Vit | NIHL | 00808470 | Micronutrients to Prevent Noise-induced Hearing Loss (Vit A-C-E, Mg) | 18-35 | All |
| GB | CPT | 01139281 | The Protective Effect of Ginkgo Biloba Extract on Cisplatin-induced Ototoxicity in Humans | >18 | All |

**A.** Non exhaustive list of preclinical assays with antioxidants (ALCAR, L-carnitine; NAC, N-acetyl cysteine; GSH, Glutathione; Vit, Vitamin complex; EBS, Ebselen; GB, Ginko biloba; FA, Ferulic acid; QTer, CoQ10 formulation; RESV, resveratrol; RA, Rosmarinic acid; MET, D-Methionine; LA, Lipoic acid; PUFAS, Poly-unsaturated fatty acids; NIT, nitrones) for the treatment of hearing loss (ARHL, age-related hearing loss; NIHL, noise-induced hearing loss; AG, aminoglycoside ototoxicity; CPT, Cisplatin ototoxicity) in different species (R, rat; M, mouse; GP, Guinea pig; CH, Chinchilla). Routes of administration: IV, intravenous; IP, intraperitoneal; PO, oral; SC, subcutaneous; IT, intratympanic. SID, once a day; BID, twice a day; ABR, auditory brainstem responses; ASSR, Auditory steady-state response; DP, Distortion product of Otoacoustic emissions; HL, Hearing loss; TS, threshold shift; HC, hair cell.

**B.** Non exhaustive list of clinical trials with antioxidants (NAC, N-acetyl cysteine; Vit, Vitamin complex; EBS, Ebselen; GB, Ginko biloba; MET, D-Methionine; LA, Lipoic acid; NIT, nitrones; STS, Sodium thiosulphate; VA, Vatiquinona) for the treatment of hearing loss (NIHL, noise-induced hearing loss; AG, aminoglycoside ototoxicity; CPT, Cisplatin ototoxicity) in patients of different age and gender. Source: <https://www.clinicaltrials.gov/ct2/home> (Jan 12, 2021).
